# Need for additional professional psychosocial and spiritual support in patients with advanced diseases in the course of specialist palliative care – a longitudinal observational study

**DOI:** 10.1186/s12904-021-00880-6

**Published:** 2021-11-25

**Authors:** Anneke Ullrich, Holger Schulz, Sven Goldbach, Wiebke Hollburg, Annette Rommel, Marten Müller, Denise Kirsch, Katrin Kopplin-Förtsch, Julia Messerer, Louise König, Frank Schulz-Kindermann, Carsten Bokemeyer, Karin Oechsle

**Affiliations:** 1 Palliative Care Unit, Department of Oncology, Hematology and BMT, University Medical Center Eppendorf, Martinistrasse 52, 20246 Hamburg, Germany; 2 Department of Medical Psychology, University Medical Center Eppendorf, Hamburg, Germany; 3 Specialist Outpatient Palliative Care Team “PalliativPartner Hamburg GbR”, Hamburg, Germany; 4 Specialist Outpatient Palliative Care Team “Das Palliativteam”, Hamburg, Germany; 5 Palliative Care Ward, Asklepios Hospital Rissen, Hamburg, Germany; 6 Specialist Outpatient Palliative Care Team “PCT Hamburg-West”, Hamburg, Germany; 7 Palliative Care Ward, Agaplesion Diakonie-Hospital, Hamburg, Germany

**Keywords:** Palliative care, Support needs, Specialist palliative care, Psychosocial needs, Spiritual needs

## Abstract

**Background:**

We investigated the need for additional professional support and associated factors in patients (pts) at initiation and in the course of in- and outpatient specialist palliative care (I-SPC/O-SPC).

**Methods:**

Pts entering an urban SPC network consecutively completed questionnaires on psychosocial/spiritual problems and support needs within 72 h (T0) as well as within the first 6 weeks (T1) of SPC. Hierarchical linear regression analysis was used to investigate the impact of sociodemographic / disease-related variables, psychological / physical burden, social support, and SPC setting on the extent of support needs.

**Results:**

Four hundred twenty-five pts (70 years, 48% female, 91% cancer, 67% O-SPC) answered at T0, and 167 at T1. At T0, main problems related to transportation, usual activities, and dependency (83–89%). At T1, most prevalent problems also related to transportation and usual activities and additionally to light housework (82–86%). At T0, support needs were highest for transportation, light housework, and usual activities (35–41%). Cross-sectional comparisons of SPC settings revealed higher problem scores in O-SPC compared to I-SPC at T0 (*p* = .039), but not at T1. Support need scores were higher in O-SPC at T0 (*p* < .001), but lower at T1 (*p* = .039). Longitudinal analyses showed a decrease of support need scores over time, independent from the SPC setting. At T0, higher distress (*p* = .047), anxiety/depression (*p* < .001), physical symptom burden (*p* < .001) and I-SPC (*p* < .001) were associated with higher support need scores (at T1: only higher distress, *p* = .037).

**Conclusion:**

Need for additional professional psychosocial/spiritual support was identified in up to 40% of pts. with higher need at the beginning of O-SPC than of I-SPC. During SPC, this need decreased in both settings, but got lower in O-SPC than in I-SPC over time. Support need scores were not only associated with psychological, but also physical burden.

**Supplementary Information:**

The online version contains supplementary material available at 10.1186/s12904-021-00880-6.

## Background

Palliative care aims to address complex problems and needs of patients including physical, psycho-spiritual, and socio-cultural aspects [1]. For cancer patients, still representing the major patient group receiving palliative care in Western countries, professional support in coping with tasks, accepting the disease, generating strength, feeling trust, strengthening the sense of control and other psychosocial and spiritual needs is considered as part of standard patient care [2, 3]. However, international experts complain that “the multiple and varying needs of patients are still not being met adequately as part of routine cancer care” [4]. Various studies underline this complaint, demonstrating high proportions of unmet needs (50–90%) in patients with cancers across all stages and during the whole disease trajectory [5–8]. It has also been shown that non-cancer patients present with similar main needs compared to a matched cohort of cancer patients, although there were some differences in quality, but not quantity of physical symptom burden [9].

Clinical studies showed that timely inclusion of specialist palliative care (SPC) can not only improve quality of life or symptom burden, but is also associated with a better addressing of patients’ needs, especially concerning information and care planning [10]. Psychosocial and spiritual interventions can successfully meet the complex psychosocial and spiritual needs of patients with advanced diseases [11–14].

SPC is offered in inpatient (I-SPC) and outpatient settings (O-SPC). Usually, the SPC setting is chosen according to patients’ individual wishes and needs. However, there are various factors reducing the probability that an outpatient care setting can address the patients’ needs adequately, e.g. patients living alone at home without any family caregivers [15], patients’ age or multi-morbidity [16]. In Germany, I-SPC was established as a billable service in 2005, and O-SPC has become prescribable by law in 2007 [17]. In contrast to the multi-professional principles of SPC in general and to the setting of I-SPC, O-SPC only comprises bi-professional care by specialized physicians and nurses, because the services of other professions such as psychologists or therapists are not covered by health insurance [7, 18]. This contrast becomes particularly manifest when patients are discharged to home care when the psychological and psycho-oncological care is interrupted [7, 19]. In daily practice, voluntary hospice workers often provide home-based psychosocial and spiritual care, while inclusion of professional support is rare and heterogeneous. This might indicate a possible care deficit, especially because up to 50% of terminally ill patients in home settings suffer from mental disorders [20].

The urban area of Hamburg was the first region in Germany that had established a complete and comprehensive network of I-SPC and O-SPC. Therefore, Hamburg can be regarded as a prime example for multi-institutional SPC networks. In this setting it is promising to evaluate if such a comprehensive SPC network can adequately address the complex problems and needs of patients with advanced diseases and terminal illnesses.

The main aim of the present study was to explore problems related to psychosocial and spiritual problems of patients at initiation and during SPC and to identify their need for additional professional support in these domains. Secondary aims concerned possible differences between patients entering I-SPC versus O-SPC. Further, we investigated the impact of sociodemographic and disease-related variables, physical symptom burden, distress, anxiety/depression and SPC setting on the extent of patients’ need for additional professional support.

## Methods

This prospective, observational longitudinal multicenter study was carried out in Hamburg, Germany. In a 12 months’ period between June 2017 and July 2018, patients were consecutively enrolled in six SPC services of an urban network, including three SPC home care services (O-SPC) and three SPC wards (I-SPC). Within 72 h after first admission, patients were recruited by trained staff of the services.

Inclusion criteria were being older than 18 years, suffering from an advanced, life-limiting illness (cancer and non-cancer), and entering in- or outpatient SPC for the first time. The participating centers cared mainly for patients with advanced cancer resulting in an expectable underrepresentation of non-cancer patients. However, these patients represent a typical cohort during SPC in Europe where up to 90% of SPC patients suffer from oncological diseases [21]. Previous studies also suggest that the problems and needs are similar between cancer and non-cancer patients [9, 22]. However, two studies comparing cancer and non-cancer patients, suggest that non-cancer patients present with lower functional status when first entering SPC [22, 23]. Exclusion criteria were cognitive or language problems hampering informed consent and/or answering questionnaires, acute physical or psychological crisis entailing the risk that study participation would significantly increase patients’ burden, and patients’ imminent death. Reasons for study exclusion or non-participation were systematically documented.

Self-report questionnaires were used for data collection, which were handed out personally by staff of the SPC services. Patients’ assistance for answering questionnaires was allowed upon request. Baseline data (T0) were collected within 72 h after admission to gain information about the patients’ situation at initiation of SPC. Follow-up measurements were scheduled as follows: As long as patients stayed in the same SPC service, questionnaires were administered every 4 weeks during ongoing SPC. Additionally, patients received a questionnaire before being transitioned to other SPC services or to non-SPC settings. The first questionnaire returned during the first 6 weeks of SPC served as follow-up (T1). Beyond this timeframe, further follow-ups were assessed according to the study protocol, but were not included in the current analysis due to limited sample sizes.

The ethical committee of the General Medical Council of Hamburg has approved the study protocol (PV5062). Written informed consent was obtained before study participation.

### Measurements

#### Outcome measure

Problems and needs for additional professional support were measured by an adapted version of the Problems and Needs in Palliative Care Questionnaire – Short Version (PNPCQ-sv) [24, 25]. With permission of its authors, the questionnaire was professionally translated to German language, and comprehensibility was tested in a small convenience sample. In order to focus on psychosocial and spiritual issues, the adapted version omitted physical symptoms. The included 22 items comprised aspects of daily activities, autonomy, need of information, and social, psychological, spiritual and financial issues, which were considered as psychosocial and spiritual issues in the broadest sense.

Two independent sum scores were calculated: the “Extent of psychosocial and spiritual problems” score (short: problem score) and the “Extent of need for additional professional support” (short: support needs score). Each score ranges from 0 to 22 with higher values reflecting a higher number of problems or support needs. Scores for respondents with ≤4 missing items were calculated by imputing the mean score for the missing items based on items completed by that individual. In case of > 4 items (20%), a score was not calculated for that individual. Respondents for which the problem score was not calculated were 9 (2.1%) at T0, and 3 (1.8%) at T1. Likewise, the support needs score was not calculated for 80 respondents (18.8%) at T0 and 27 (16.2%) at T1.

#### Potential predictor variables

The distress thermometer (DT) was used to assess psychological distress within the last week on an 11-point analogue scale. Clinically relevant distress with need of professional psychological support is indicated by a cut-off value of ≥5 [26, 27]. The DT includes a problem list with 21 physical symptoms that can be classified to contribute to psychological distress or not. We used the sum score of distressing physical symptoms (“physical symptom count”, 0–21) to estimate the range of patients’ physical symptoms (see supplemental material 1).

The PHQ-4 including a two-item depression scale (PHQ-2), and a two-item anxiety scale (GAD-2) was used for measurement of depressive and anxiety symptoms [28].

In addition, patients reported on sociodemographic characteristics (i.e. age, marital status, educational level, living environment), as well as disease-related data (i.e. primary disease, previous nursing situation).

### Statistical analyses

We performed descriptive analyses to examine study population characteristics and to describe patients’ psychosocial and spiritual problems and their need for additional professional support.

Data of patients admitted to I-SCP vs. O-SPC at initiation of SPC (T0) and at follow-up during SPC (T1) were compared cross-sectional using chi-square-tests (Fisher’s exact test if expected values in any cell were below 5) and two-sample t-tests (two-tailed).

The problem scores and support need score at T0 were compared with the respective scores at T1 using repeated measures ANOVA (RM-ANOVA) with setting (O-SPC vs. I-SPC) as a main factor. As measures of effect, we calculated partial eta-squared (ŋ_p_^2^; small = 0.01, medium = 0.06, large = 0.14).

Two hierarchical linear regression models (enter method) were used to investigate the impact of sociodemographic and disease-related variables, psychological and physical burden, and SPC setting on the extent of patients’ need for additional support. Support needs scores at T0 and at T1 were defined as dependent variables, respectively. Sociodemographic variables were added in step 1 (Model 1), disease-related variables at step 2 (Model 2), proxy-variables for social support at step 3 (Model 3), physical burden at step 4 (Model 4), and variables reflecting psychological burden at step 5 (Model 5). Categorical predictor variables were dichotomized. Examination of correlations among the predictor variables revealed no problems with multicollinearity. Missing data were handled using the list wise deletion method.

All significance tests were two-tailed using a significance level of α < .05. Analyses were completed using SPSS software version 25.0 (IBM, 2017).

## Results

### Patient recruitment and characteristics

**Fig. 1:**
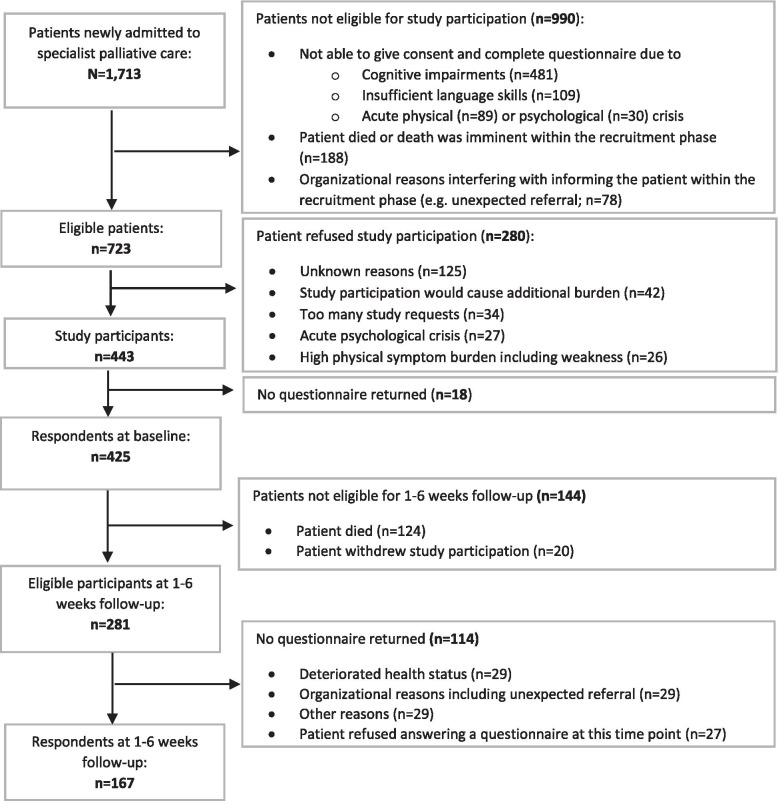
Recruitment process and patient cohort development

During recruitment, 1713 patients were admitted to the six participating services. Of these, 713 (42%) were eligible for study inclusion, and 443 were willing to participate (61%). Among 425 who returned the baseline questionnaires (T0), 285 were admitted to O-SPC (67%) and 140 to I-SPC (33%). At follow-up during SPC (T1), 167 patients (39%) answered a questionnaire. At this time point, 130 (78%) were treated in O-SCP and 37 (22%) in I-SPC (Fig. 1).

Overall, at initiation of SPC (T0) 52% of 425 patients were male (mean age 69.7 ± 12.5 years) and 91% suffered from cancer. Patients admitted to O-SPC were significantly younger (*p* < .001) and had needed less intensive nursing prior to SPC (*p* = .008) than in I-SPC (Table 1).

**Table 1:** Patient characteristics at baseline (*N* = 425)

	Whole sample (***N*** = 425)	O-SPC (***N*** = 285)	I-SPC (***N*** = 140)	
	n (%)	n (%)	n (%)	***p***
Age, M (SD)	69.7 (12.5)	71.4 (11.3)	66.2 (14.0)	**<.001** ^a^
Age groups				
≤ 50	28 (6.6)	15 (5.3)	13 (9.3)	.013 ^b^
51–60	65 (15.3)	35 (12.3)	30 (21.4)	
61–70	110 (25.9)	71 (24.9)	39 (27.9)	
71–80	136 (32.0)	99 (34.7)	37 (26.4)	
≥ 81	86 (20.2)	65 (22.8)	21 (15.0)	
Gender				
Male	220 (51.9)	146 (51.4)	74 (52.9)	.779 ^b^
Female	205 (48.1)	138 (48.6)	66 (47.1)	
Primary disease				
Gastrointestinal cancer	104 (24.5)	63 (22.1)	41 (29.3)	.209 ^b^
Cancer of the respiratory system	78 (18.4)	60 (21.1)	18 (12.9)	
Urogenital and breast cancer	136 (32.0)	90 (31.6)	46 (32.9)	
Other malignancies	72 (16.9)	47 (16.5)	25 (17.9)	
Non-malignant diseases	35 (8.2)	25 (8.8)	10 (7.1)	
Nationality				
German	395 (95.9)	260 (94.5)	135 (98.5)	.055 ^b^
Religious confession				
Yes	235 (57.5)	150 (54.9)	85 (62.5)	.14 ^b^ 5
Family status				
Single	80 (18.9)	46 (16.2)	34 (24.3)	.105 ^b^
Married, life partnership	211 (49.8)	143 (50.4)	68 (48.6)	
Divorced, widowed	133 (31.4)	95 (33.5)	38 (27.1)	
Children				
Yes	302 (71.4)	205 (72.2)	97 (69.8)	.608 ^b^
Living environment				
Living alone	135 (32.3)	90 (32.4)	45 (32.1)	.427 ^b^
Living alone, but family nearby	48 (11.5)	28 (10.1)	20 (14.3)	
Living with family	235 (56.2)	160 (57.6)	75 (53.6)	
Education				
High school (12–13 years)	129 (31.2)	81 (29.5)	48 (34.5)	.566 ^b^
Junior high school (10 years)	117 (28.3)	79 (28.7)	38 (27.3)	
Elementary school (≤ 9 years)	168 (40.6)	115 (41.8)	53 (38.1)	
Previous nursing situation				
No nursing	78 (19.3)	41 (15.2)	37 (27.4)	**.008** ^b^
By relatives only	151 (37.3)	99 (36.7)	52 (38.5)	
Nursing service only	116 (28.6)	88 (32.6)	28 (20.7)	
Nursing service and relatives	60 (14.8)	42 (15.6)	18 (13.3)	

Significant group differences are marked in bold

*Abbreviations*: *O-SPC* outpatient specialist palliative care, *I-SPC* inpatient specialist palliative care, *pts* patients

^a^ T-test (two-tailed), ^b^ Chi^2^-Test

### Cross-sectional analyses of problems at initiation and during SPC

At initiation of SPC (T0), the five most prevalent problems were “transportation” (88.9%), “difficulties in continuing the usual activities” (88.9%), “being dependent of others” (82.6%), “doing light housework” (82.1%), and “body care, washing, dressing or toilet” (70.4%). Problems in the psychological and spiritual domains were reported less often, but were still indicated by 44 to 69% (psychological issues) and 45 to 55% (spiritual issues) of patients. A significant difference between the O-SPC and I-SPC group was observed in six of 22 given problems. Except for one aspect, these problems were more frequent in O-SPC, resulting in a higher mean problem score in the O-SPC vs. I-SCP group (11.7 vs. 11.1, *p* = .039).

During SPC (T1), the five most common problems were “transportation” (85.5%), “difficulties in continuing the usual activities” (85.3%), “doing light housework” (81.7%), “difficulty coping with the unpredictability of the future” (67.3%), and “body care, washing, dressing or toilet” (67.1%). Again, psychological (39 to 67%) and spiritual problems (37 to 58%) were indicated less often. Problem scores did not differ significantly between patients receiving O-SPC vs. I-SPC at this point of time (*p* = .278.). However, patients receiving I-SPC more often reported problems regarding “transportation” (*p* = .049) and “finding others not receptive to talking about the disease” (*p* = .003, Table 2).

**Table 2:** Cross-sectional comparisons of patients’ psychosocial and spiritual problems during O-SPC vs. I-SPC

	At initiation of SPC (T0) (*N* = 425)				During SPC (T1) (*N* = 167)			
	Whole sample (*N* = 425)	O-SPC (*N* = 285)	I-SPC (*N* = 140)		Whole sample (*N* = 167)	O-SPC (*N* = 125)	I-SPC (*N* = 42)	
Psychosocial and spiritual problems (PNPCQ-sv)	n (%) yes	n (%) yes	n (%) yes	p ^a^	n (%) yes	n (%) yes	n (%) yes	p ^a^
Daily activities								
Body care, washing, dressing, or toilet	299 (70.4)	204 (72.9)	95 (67.9)	.286	112 (67.1)	84 (67.2)	28 (66.7)	.949
Transportation	378 (88.9)	261 (92.9)	117 (84.2)	**.005**	141 (85.5)	109 (76.2)	32 (88.6)	**.049**
Doing light housework	349 (82.1)	244 (87.1)	105 (76.1)	**.004**	134 (81.7)	102 (82.9)	32 (78.0)	.484
Autonomy								
Difficulties in continuing the usual activities	378 (88.9)	256 (91.1)	122 (88.4)	.382	139 (85.3)	105 (85.4)	34 (85.0)	.955
Difficulty to give tasks out of hands	232 (54.6)	154 (55.0)	78 (57.4)	.650	88 (53.3)	62 (50.0)	26 (63.4)	.136
Being dependent of others	351 (82.6)	235 (82.5)	116 (83.5)	.798	130 (30.6)	97 (77.6)	33 (80.5)	.679
Experiencing loss of control over one’s life	272 (64.0)	180 (64.1)	92 (68.1)	.412	93 (56.0)	65 (52.4)	28 (66.7)	.108
Social issues								
Problems in the relationship with life companion	85 (20.0)	65 (23.2)	20 (14.6)	**.040**	24 (14.5)	15 (12.1)	9 (21.4)	.137
Difficulties in talking about the disease with life companion	128 (30.1)	94 (33.5)	34 (24.6)	.066	34 (20.5)	24 (19.2)	10 (24.4)	.475
Finding it difficult to talk about the disease, because of not wanting to burden others	225 (52.9)	156 (55.9)	69 (50.7)	.320	67 (40.1)	49 (39.2)	18 (42.9)	.676
Finding others not receptive to talking about the disease	153 (36.0)	105 (37.2)	48 (35.0)	.661	46 (28.0)	27 (22.0)	19 (46.3)	**.003**
Difficulties in finding someone to talk to	145 (34.1)	100 (35.5)	45 (32.6)	.564	45 (27.4)	33 (26.8)	12 (29.3)	.762
Psychological issues								
Depressed mood	188 (44.3)	131 (46.5)	57 (41.3)	.319	64 (38.6)	48 (38.7)	16 (38.1)	.944
Difficulty coping with the unpredictability of the future	291 (68.5)	200 (71.4)	91 (66.4)	.296	111 (67.3)	84 (67.7)	27 (65.9)	.823
Difficulties to show emotions	212 (49.9)	143 (50.7)	69 (50.0)	.891	71 (43.6)	49 (39.8)	22 (55.0)	.093
Spiritual issues								
Difficulties to be engaged usefully	206 (48.5)	144 (52.0)	62 (44.9)	.175	86 (52.4)	66 (53.2)	20 (50.0)	.722
Difficulties to be of avail of others	233 (54.8)	160 (58.0)	73 (53.3)	.366	96 (58.5)	68 (55.3)	28 (68.3)	.143
Difficulties concerning the meaning of death	192 (45.2)	138 (49.8)	54 (39.7)	.053	60 (37.3)	46 (38.0)	14 (35.0)	.732
Difficulties to accept the disease	233 (54.8)	168 (59.2)	65 (47.1)	**.019**	83 (50.9)	61 (49.6)	22 (55.0)	.522
Financial problems								
Extra expenditures because of the disease	125 (29.4)	85 (30.5)	40 (29.2)	.791	40 (27.4)	29 (23.8)	11 (27.5)	.635
Loss of income because of the disease	71 (16.7)	39 (14.1)	32 (23.7)	**.015**	28 (17.5)	20 (16.7)	8 (20.0)	.631
Need of information								
Insufficient information, e.g. about the disease and its treatment, aids and agencies that can provide help, alternative healing methods.	140 (32.9)	105 (37.9)	35 (25.4)	**.011**	38 (23.5)	27 (22.1)	11 (27.5)	.487
Score “Extent of psychosocial and spiritual problems” (0–22)c, M (SD)	11.7 (4.9)	11.7 (4.9)	11.1 (4.0)	**.039** ^**b**^		10.5 (4.7)	11.2 (4.9)	.278 ^**b**^

Significant group differences are marked in bold

*Abbreviations*: *SPC* specialist palliative care; pts., patients, *O-SOC* outpatient specialist palliative care, *I-SPC* inpatient specialist palliative care, *PNPCQ-sv* Problems and Needs in Palliative Care Questionnaire – Short Version

^a^ Chi^2^-Test; ^b^ T-test (two-tailed)

### Cross-sectional analyses of need for additional professional support at initiation and during SPC

At T0, the five most common needs for additional professional support were “transportation” (40.5%), “doing light housework” (38.4%), “difficulties in continuing the usual activities” (35.2%), “difficulty coping with the unpredictability of the future” (29.9%), and “being dependent of others” (28.9%). Need for additional professional support was indicated by 15.3 to 29.9% of patients concerning psychological problems, and 18.4 to 24.7% concerning spiritual problems. A significant difference between the O-SPC and I-SPC group was observed in nine of 22 given needs, including all spiritual aspects. These needs were consistently more prevalent in O-SPC compared to I-SPC. Additionally, the mean needs score showed to be significantly higher in patients receiving O-SPC (6.4 vs. 4.1; *p* < .001).

At T1, need for additional professional support had decreased in most aspects. Among 167 patients, the five most frequent needs for more support related to “transportation” (24.0%), “doing light housework” (19.9%), “being dependent of others” (19.7%), “difficulties in continuing the usual activities” (19.6%), and “difficulty coping with the unpredictability of the future” (13.2%). Need for additional support was reported by 3.2 to 13.2% of patients for psychological problems, and by 9.0 to 11.8% for spiritual problems. During SPC (T1), the mean support need score was significantly lower in O-SPC compared to I-SPC (1.7 vs 3.2; *p* = .038). At the level of single needs, “difficulties to accept the disease” was experienced differently across SPC settings with patients in O-SPC reporting less need for support (7.0% vs. 18.9%, *p* = .034; Table 3).

**Table 3:** Cross-sectional comparisons of patients’ need for additional professional psychosocial and spiritual support during O-SPC vs. I-SPC

	At initiation of SPC (T0) (*N* = 425)				During SPC (T1) (*N* = 167)			
	Whole sample (*N* = 425)	O-SPC (*N* = 285)	I-SPC (*N* = 140)		Whole sample (*N* = 167)	O-SPC (*N* = 125)	I-SPC (*N* = 42)	
**Need for additional psychosocial and spiritual support (PNPCQ-sv)**	n (%) yes	n (%) yes	n (%) yes	p	n (%) yes	n (%) yes	n (%) yes	p
Daily activities:								
Body care, washing, dressing, or toilet	121 (28.5)	96 (34.7)	25 (18.0)	**<.001** ^a^	19 (12.0)	13 (11.0)	6 (15.0)	.503 ^a^
Transportation	172 (40.5)	121 (45.0)	51 (38.6)	.228 ^a^	35 (24.0)	23 (21.5)	12 (30.8)	.245 ^a^
Doing light housework	163 (38.4)	120 (45.1)	43 (32.8)	**.019** ^a^	29 (19.9)	20 (18.0)	9 (25.7)	.320 ^a^
Autonomy:								
Difficulties in continuing the usual activities	150 (35.2)	99 (37.8)	51 (38.1)	.958 ^a^	28 (19.6)	17 (16.2)	11 (28.9)	.089 ^a^
Difficulty to give tasks out of hands	85 (20.0)	60 (22.0)	25 (18.8)	.460 ^a^	12 (8.0)	6 (5.5)	6 (15.0)	.057 ^a^
Being dependent of others	123 (28.9)	85 (31.3)	38 (28.6)	.582 ^a^	28 (19.7)	18 (17.4)	10 (26.3)	.232 ^a^
Experiencing loss of control over one’s life	114 (26.8)	78 (28.5)	36 (26.9)	.735 ^a^	14 (9.7)	8 (7.6)	6 (15.4)	.162 ^a^
Social issues:								
Problems in the relationship with life companion	38 (8.9)	29 (10.4)	9 (6.6)	.207 ^a^	1 (0.6)	1 (0.8)	0 (0)	1.000 ^b^
Difficulties in talking about the disease with life companion	59 (13.9)	46 (19.0)	13 (10.9)	.051 ^a^	2 (1.3)	1 (0.8)	1 (2.4)	.448 ^b^
Finding it difficult to talk about the disease, because of not wanting to burden others	96 (22.6)	72 (30.0)	24 (19.5)	**.032** ^a^	7 (4.6)	3 (2.7)	4 (9.8)	.083 ^b^
Finding others not receptive to talking about the disease	54 (12.7)	43 (17.9)	11 (9.3)	**.033** ^a^	4 (2.6)	2 (1.8)	2 (5.0)	.280 ^b^
Difficulties in finding someone to talk to	78 (18.4)	54 (22.4)	24 (20.3)	.655 ^a^	3 (1.9)	1 (0.9)	2 (4.9)	.173 ^b^
Psychological issues:								
Depressed mood	75 (17.6)	57 (23.2)	18 (15.1)	.075 ^a^	5 (3.2)	4 (3.5)	1 (2.4)	1.000 ^b^
Difficulty coping with the unpredictability of the future	127 (29.9)	88 (35.8)	39 (30.5)	.304 ^a^	20 (13.2)	12 (10.7)	8 (20.5)	.120
Difficulties to show emotions	65 (15.3)	47 (19.3)	18 (14.0)	.199 ^a^	7 (4.5)	4 (3.4)	3 (7.3)	.378 ^b^
Spiritual issues:								
Difficulties to be engaged usefully	78 (18.4)	59 (24.6)	19 (15.6)	**.049** ^a^	13 (9.0)	10 (9.3)	3 (8.1)	1.000 ^b^
Difficulties to be of avail of others	85 (20.0)	66 (28.0)	19 (15.6)	**.009** ^a^	17 (11.8)	10 (9.3)	7 (19.4)	.101 ^a^
Difficulties concerning the meaning of death	94 (22.1)	72 (30.4)	22 (18.6)	**.018** ^a^	16 (10.3)	10 (8.7)	6 (15.0)	.259 ^a^
Difficulties to accept the disease	105 (24.7)	88 (36.5)	17 (14.4)	**<.001** ^a^	15 (9.9)	8 (7.0)	7 (18.9)	**.034** ^a^
Financial problems:								
Extra expenditures because of the disease	66 (15.5)	48 (21.0)	18 (14.8)	.156 ^a^	9 (5.8)	5 (4.3)	4 (10.5)	.225 ^b^
Loss of income because of the disease	41 (9.6)	28 (12.1)	13 (10.8)	.732 ^a^	9 (5.8)	6 (5.1)	3 (7.9)	.690 ^b^
Need of information:								
Insufficient information, e.g. about the disease and its treatment, aids and agencies that can provide help, alternative healing methods.	116 (27.3)	90 (38.5)	26 (21.5)	**.001** ^a^	15 (9.6)	9 (7.6)	6 (15.8)	.133
Score “Extent of need for additional professional support” (0–22) M (SD)		6.4 (6.5)	4.2 (4.1)	**<.001** ^**c**^		1.7 (2.9)	3.2 (4.0)	**.038** ^**c**^

Significant group differences are marked in bold

*Abbreviations*: *SPC* specialist palliative care; pts., patients, *O-SOC* outpatient specialist palliative care, *I-SPC* inpatient specialist palliative care, *PNPCQ-sv* Problems and Needs in Palliative Care Questionnaire – Short Version

^a^ Chi2-Test; ^b^ Fisher’s Exact Test; ^c^ T-test (two-tailed); d Higher scores reflect a greater number of needs for which additional professional psychosocial and spiritual support was indicated

### Longitudinal analyses of problems and need for additional professional support

Comparing problem scores at T0 with T1, no significant effects were detected by RM-ANOVA. Analyzing the course of the support needs scores, significant improvement could be demonstrated over time (large time effect: *p* < .001, ŋ_p_^2^ = .106), meaning that less needs were reported to be unmet. However, we found no significant effects of the setting (O-SPC/I-SPC) or interaction effects of time and setting (Table 4).

**Table 4:** Comparison of the total scores of psychosocial and spiritual problems as well as need for additional professional support assessed at initiation of specialist palliative care (T0) and at follow-up (T1) using repeated measures ANOVA

	O-SPC	I-SPC	Main Effect Time				Main Effect Setting				Setting x Time Interaction			
	M (SD)	M (SD)	df	F	p	ŋ_**p**_^**2**^	df	F	p	ŋ_**p**_^**2**^	df	F	p	ŋ_**p**_^**2**^
**Score “Extent of psychosocial and spiritual problems” (0–22)**														
At initiation of SPC (T0)	11.2 (4.9)	10.8 (3.8)	1	.797	.373	.005	1	.000	.996	.000	1	.700	.404	.004
During SPC (T1)	10.5 (4.7)	10.8 (4.7)												
**Score “Extent of need for additional professional support” (0–22)**														
At admission (T0)	4.8 (5.8)	3.6 (3.5)	1	13.158	**<.001**	.106	1	.158	.692	.001	1	2.087	.151	.018
During SPC (T1)	1.9 (3.3)	2.4 (3.8)												

Significant *p*-values are marked in bold

*Abbreviations*: *SPC* specialist palliative care, *M* mean, *SD* standard deviation, *d.f.* degrees of freedom, *F* F-statistic, *ŋ*_*p*_^*2*^ partial eta square, *p* probability of type I error

### Potential predictors for additional professional psychosocial or spiritual support needs at initiation and during SPC

Predictors for the extent of support needs at both time points were identified by hierarchical linear regression analyses.

At T0, variables of step 1 to 3 (sociodemographic, disease-related, and proxy-variables for social support) only explained up to 5% of the variance in the support needs score, and SPC setting showed to be the single predictor. Physical burden, tested in step 4, explained an additional 25% of variance. Psychological factors, tested in step 5, explained an additional 10% of variance. Higher distress (*p* = .047) and higher level of anxiety/depressive symptoms (*p* < .001) were associated with the support needs score. Additionally, SPC setting (*p* < .001) and physical burden (*p* < .001) remained significantly associated in this final model.

At T1, variables tested in step 1 to 3 only explained up to 8% of the variance with primary disease being the only significant predictor. Physical burden, tested in step 4, explained an additional 4% of variance. Psychological factors, tested in step 5, explained an additional 7% of variance when controlling the other factors. Higher distress (*p* = .037) was associated with the support needs score, and neither of the other factors remained significantly associated in the final model (Table 5).

**Table 5:** Summary of hierarchical regression analysis for variables predicting the extent of need for additional professional psychosocial and spiritual support when first entering SPC (T0) and during SPC (T1)

Predictor variables^a^	Model 1 ***ß***	Model 2 ***ß***	Model 3 ***ß***	Model 4 ***ß***	Model 5 ***ß***
***Score “Need for additional professional support at initiation of SPC” (T0, N = 314***^*b*^***)***					
Age	−.01	−.03	−.05	−.02	−.02
Gender (0, female; 1, male)	.01	.15	.01	.02	−.03
Primary disease (0, non-cancer; 1, cancer)		−.12	−.11	−.10	−.04
SPC setting (0, inpatient; 1, outpatient)		.17**	.17**	.21***	.17***
Care site prior SPC (0, at home 1; nursing home/hospital)		−.04	−.04	−.04	−.03
Having children (0, no; 1, yes)			−.03	−.01	−.01
Living situation (0, living alone; 1, with relatives or relatives nearby)			−.08	−.12*	−.09
Physical symptom count (0–21)				.51***	.33***
Distress (DT; 0–10)					.10*
Anxiety/depressive symptoms (PHQ-4; 0–12)					.33***
*R*^2^	.00	.04	.05	.30	.40
Adjusted *R*^2^	−.01	.03	.03	.28	.38
*F* for change in *R*^2^	.04	4.4	1.4	109.73	26.02
***Score “Need for additional professional support during SPC (T1, N = 129***^*b*^***)***					
Age	−.11	−.13	−.12	−.14	−.10
Gender (0, female; 1, male)	.08	.09	.08	.08	.02
Primary disease (0, non-cancer; 1, cancer)		−.18*	−.19*	−.19*	−.14
SPC setting (0, inpatient; 1, outpatient)		−.16	−.15	−.12	−.10
Care site prior SPC (0, at home 1; nursing home/hospital)		−.08	−.08	−.07	−.04
Having children (0, no; 1, yes)			−.03	−.01	.03
Living situation (0, living alone; 1, with relatives or relatives nearby)			−.03	−.07	−.08
Physical symptom count (0–21)				.21*	1.0
Distress (DT; 0–10)					2.0*
Anxiety/depressive symptoms (PHQ-4; 0–12)					.19
*R*^2^	.02	.08	.08	.12	.19
Adjusted *R*^2^	.00	.04	.03	.06	.13
*F* for change in *R*^2^	1.04	2.7	.18	5.67	5.30

Hierarchical linear regression analysis (enter method). Step 1: demographic variables (age, sex), step 2: care-related variables (primary disease, SPC setting, care site prior SPC); step 3: proxy-variables for social support (children, living situation), step 4: Physical burden (physical symptom count), step 5: variables reflecting psychological burden (distress, anxiety/depressive symptoms)

*Abbreviations*: *ß* Standardized regression coefficients, *SPC* specialist palliative care, *DT* Distress Thermometer, *PHQ-4* Patient Health Questionnaire 4-item version

^a^ All potential predictor variables were measured at onset of specialist palliative care (T0), ^b^ Reduced sample size due to missing values (analyzed by listwise deletion)

* *p* <. 05

** *p* < .01

*** *p* < .001

## Discussion

This prospective longitudinal study evaluated psychosocial and spiritual problems of patients receiving I-SPC or O-SPC, their need for additional professional psychosocial and spiritual support and predictors for a higher number of support needs.

### Psychosocial and spiritual problems

The most prevalent problems of patients at initiation of SPC were problems in the dimension of daily activities. During SPC, four of the five most frequent problems were still related to daily activities. However, with “difficulty coping with the unpredictability of the future” as fourth frequent problem, a psychological aspect gained on impact. In line, in a previous German study patients receiving O-SPC most commonly reported problems concerning daily activities (fatigue, getting around, eating, bathing/dressing) [29]. The impact of such impairments is also emphasized by a study reporting that problems in the area of functioning were most frequently complained by patients receiving SPC [30]. In our study, spiritual problems were less frequently identified than psychological problems, but still concerned about 40% of patients at both assessments. This matches with previous studies reporting an importance of spirituality / spiritual aspects during palliative care in about 30–40% of patients [31].

Comparing O-SPC and I-SPC, problems tended to be more frequent in the O-SPC group at initiation of SPC, which also reflected in significantly higher problem scores. Despite these differences, patients from both settings showed relevant problems requiring multi-professional care. In contrast, the setting did not influence problem scores at follow-up during SPC; however, prevalence of two single problems (“transportation”, “finding others not receptive to talking about the disease”) was significantly higher in the I-SPC group. In a retrospective study comparing O-SPC and I-SPC, O-SPC patients had worse function and higher need for care planning and family support [31, 32].

Longitudinal analyses of problem scores showed no significant effects of time and setting. This might not be surprising as the patients’ advanced diseases progress over time, and SPC could only maintain stability of symptoms and problem burden.

### Need for additional professional psychosocial and spiritual support

The need for additional support was lower than the extent of the corresponding problems at both assessments indicating that patients might not expect or want professional support for every problem they perceive, especially regarding psychological and spiritual problems. While some patients require deeper psychological and spiritual support (ranging from approximately 10 to 40%), others might prefer to cope with such problems themselves. Additionally, some problems might be addressed by informal support, e.g. family caregivers, or patients might not be able to feel and express their need for additional professional support.

Reviewing the relative prevalence of needs reported in our study, need for additional support related to daily activities was most common both at initiation and during SPC: At initiation of SPC, the three most common needs for additional support concerned daily activities, followed by two psychological issues regarding the difficulty to cope with the unpredictability of the future and being dependent of others. During SPC, the need for additional support decreased in most aspects with the same needs representing the “top five”. Literature on patients’ support needs mainly relates on cancer patients [8, 30, 33–42]. These studies also demonstrate that daily living, practical support, information and emotional/psychological support consistently represent the most prevalent unmet support needs [8, 30, 33–42]. In line with the results from our study, a repeatedly mentioned psychological problem causing support need is the unpredictably of future [8, 38, 41].Additional need for professional spiritual support was lower with about 20% at initiation of SPC and about 10% during follow-up indicating that such support is required by a subgroup of patients. However, studies strengthen the lack of spiritual support even during SPC in a still relevant number of patients [9].

These findings are both understandable and consistent with Maslow’s hierarchy of needs theory [43]. Maslow’s hierarchy of needs divides human needs in five categories from low to high (like a pyramid), starting with basic physiological demands. The theory proposes that if needs of a certain level are satisfied, the next higher need emerges and the pursuit of a higher level of needs becomes the driving force for behavior. In our study, the highest needs for additional professional support related to the more basic needs, like daily activities and transportation, which may have to be met for patients to feel safe enough to attend to psychological and spiritual needs. However, our findings also indicated that higher needs may already be important although lower-level may have not been met. Despite the usefulness of this theory for assessing patients’ needs in palliative care, the criticism that Maslow’s ranking of needs might be overly simplistic has to be kept in mind [44].

Comparing SPC settings, patients scored significantly higher additional support need when entering O-SPC. Interestingly, during SPC, the need for additional psychosocial and spiritual support was higher in I-SPC. However, higher support needs during inpatient compared to outpatient care in cancer patients have been demonstrated [33].

Longitudinal analyses of patients’ need for additional support showed a significant improvement over time, but without any difference between the two SPC settings. This result indicates that the two different SPC settings satisfy the needs of the referred patient cohorts, which strengthens the results of a previous study demonstrating that SPC could meet the needs of their target group [39].

### Factors associated with additional professional support needs

At initiation of SPC, higher support need scores were associated with higher physical symptom burden, higher distress, higher levels of anxiety/depression and I-SPC setting. In contrast, during SPC, higher distress revealed to be the only predictor of support needs. Previous studies have also reported significant effects of distress and problem or symptom burden on patients’ support needs [29]. In our study, sociodemographic or disease-related factors were not identified to be predictive, which is in line with previous studies that could not find any or only partial associations between sociodemographic or medical/disease-related factors in patients with advanced diseases [29, 38, 40]. Only one study including older cancer patients observed that non-white, divorced or never married patients had higher unmet social support needs [39].

### Strengths and limitations

This study has some strengths, e.g. the prospective longitudinal design, consecutive patient recruitment within an established SPC network, and systematic documentation of non-response. In addition, our study specifically evaluates the need for professional support in patients receiving SPC. Previous study mainly analyzed “unmet needs” which does not clarify if the patients expect professional or non-professional support, e.g. from family caregivers.

However, there are some limitations that have to be noticed. Our SPC network is located in an urban region and it remains unclear if these results could be transferred to patients in more rural areas.

We included aspects of daily living as psychosocial aspects when using the PNPC-sv, while some other questionnaires define them as a separate or physical category. However, the PNPC-sv is one of the two most commonly used questionnaire in needs assessment at least in cancer patients [36]. Overall, the different questionnaires used for needs assessment hamper comparability of data in general [35, 36].

In conclusion, a relevant number of problems, mainly concerning limited daily activities, should be considered in patients entering SPC. Psychological and spiritual problems could be expected more frequently in patients entering O-SPC, but this difference will be balanced during SPC, indicating that SPC services can meet professional care for basic needs, but also for higher-level needs, like psychological and spiritual needs. However, healthcare professionals should respect that patients do not expect or want professional support for all of their problems. In daily routine care, assessments should be used that allow to distinguish between problems and (unmet) needs, which are two different concepts. Patients requests for additional professional support mainly concern aspects of daily living, dependency and coping with the unpredictability of the future. Spiritual problems and need for additional professional support are less frequent than psychological. Patients’ need for additional support decreases during SPC, but does not show any difference between the two SPC settings. Patients’ support needs are associated with psychological distress and physical symptom burden, but not sociodemographic or disease-related factors.

## Supplementary Information

**Additional file 1:** **Supplemental Material 1.** Patients’ distressing physical problems during O-SPC vs. I-SPC (cross-sectional).

## Data Availability

All authors have full control over the primary data. The data are analyzed in this study are housed at the Palliative Care Unit, Department of Oncology, Hematology and BMT, University Medical Center Hamburg-Eppendorf, Martinistrasse 52, 20246 Hamburg, Germany, Prof. Dr. Karin Oechsle, corresponding author. As per the ethical committee approval, this dataset is subject to ethical restrictions, and informed written consent of study participants does not include publication of raw data. All relevant data for the conclusions are presented in the manuscript.
