# Supplementary material for: Need for additional professional psychosocial and spiritual support in patients with advanced diseases in the course of specialist palliative care – a longitudinal observational study
Source: BMC Palliat Care. 2021 Nov 25;20:182. doi: 10.1186/s12904-021-00880-6 (PMC8613968; doi:10.1186/s12904-021-00880-6)
Supplement: Supplementary file 1 — Additional file 1: Supplemental Material 1. Patients’ distressing physical problems during O-SPC vs. I-SPC (cross-sectional). [file 12904_2021_880_MOESM1_ESM.docx]

**Supplemental material 1**: Patients’ distressing physical problems during O-SPC vs. I-SPC (cross-sectional)

|  |  | At initiation of SPC (T0)  (N=425) | | | | During SPC (T1)  (N=167) | | | |
| --- | --- | --- | --- | --- | --- | --- | --- | --- | --- |
|  |  | Whole sample  (N=425) | O-SPC  (N=285) | I-SPC  (N=140) |  | Whole sample  (N=167) | O-SPC  (N=125) | I-SPC  (N=42) |  |
| **Physical (DT)** | | n (%) | n (%) | n (%) | p | n (%) | n (%) | n (%) | p |
| DT problem list: physical symptoms: | |  |  |  |  |  |  |  |  |
|  | Pain | 320 (75.3) | 216 (75.8) | 104 (74.3) | .735 ^a^ | 105 (62.9) | 79 (63.2) | 26 (61.9) | .881 ^a^ |
|  | Nausea | 227 (53.4) | 146 (51.2) | 81 (57.9) | .198 ^a^ | 82 (37.1) | 44 (35.2) | 18 (42.9) | .374 ^a^ |
|  | Fatigue | 385 (90.6) | 256 (89.8) | 129 (92.1) | .442 ^a^ | 141 (84.4) | 105 (84.0) | 36 (85.7) | .791 ^a^ |
|  | Sleep | 423 (99.5) | 156 (55.1) | 76 (54.3) | .871 ^a^ | 75 (44.9) | 58 (46.4) | 17 (40.5) | .504 ^a^ |
|  | Getting around | 379 (89.2) | 257 (90.2) | 122 (87.1) | .344 ^a^ | 132 (79.0) | 99 (79.2) | 33 (78.6) | .931 ^a^ |
|  | Bathing / dressing | 274 (64.5) | 184 (64.6) | 90 (64.3) | .955 ^a^ | 92 (55.1) | 67 (53.6) | 25 (59.5) | .504 ^a^ |
|  | Appearance | 178 (41.9) | 129 (45.3) | 49 (35.0) | **.044** ^a^ | 58 (34.7) | 43 (34.4) | 15 (35.7) | .877 ^a^ |
|  | Breathing | 235 (55.3) | 149 (52.3) | 86 (61.4) | .075 ^a^ | 75 (44.9) | 57 (45.6) | 18 (42.9) | .757 ^a^ |
|  | Mouth sores | 114 (26.8) | 76 (26.7) | 38 (27.1) | .917 ^a^ | 36 (21.6) | 27 (21.6) | 9 (21.4) | .982 ^a^ |
|  | Eating | 276 (64.9) | 193 (67.7) | 83 (59.3) | .087 ^a^ | 89 (53.3) | 70 (56.0) | 19 (45.2) | .226 ^a^ |
|  | Indigestion | 263 (61.9) | 179 (62.8) | 84 (60.0) | .575 ^a^ | 70 (41.9) | 49 (39.2) | 21 (50.0) | .220 ^a^ |
|  | Constipation | 209 (49.2) | 134 (47.0) | 75 (53.6) | .217 ^a^ | 53 (31.7) | 36 (28.8) | 17 (40.5) | .160 ^a^ |
|  | Diarrhea | 100 (23.5) | 68 (23.9) | 32 (22.9) | .819 ^a^ | 40 (24.9) | 31 (24.8) | 9 (21.4) | .658 ^a^ |
|  | Changes in urination | 148 (34.8) | 95 (33.3) | 53 (37.9) | .358 ^a^ | 36 (21.6) | 27 (21.6) | 9 (21.4) | .981 ^a^ |
|  | Fevers | 37 (8.7) | 18 (6.3) | 19 (13.6) | **.013** ^a^ | 8 (4.8) | 6 (4.8) | 2 (4.8) | 1.000 ^b^ |
|  | Skin dry/itchy | 204 (48.0) | 132 (46.3) | 72 (51.4) | .321 ^a^ | 57 (34.1) | 44 (35.2) | 13 (31.0) | .615 ^a^ |
|  | Nose dry/congested | 143 (33.6) | 84 (29.5) | 59 (42.1) | **.009** ^a^ | 41 (24.7) | 27 (21.8) | 14 (33.3) | .133 ^a^ |
|  | Tingling in hands/feet | 173 (40.7) | 98 (34.4) | 75 (53.6) | **<.001** ^a^ | 65 (38.9) | 46 (36.8) | 19 (45.2) | .332 ^a^ |
|  | Feeling swollen | 153 (36.0) | 87 (30.5) | 66 (47.1) | **.001** ^a^ | 47 (28.1) | 29 (23.2) | 18 (42.9) | **.014** ^a^ |
|  | Memory/concentration | 200 (47.1) | 127 (44.6) | 73 (52.5) | .123 ^a^ | 64 (38.3) | 44 (35.2) | 20 (47.6) | .152 ^a^ |
|  | Sexual problems | 109 (25.6) | 73 (25.7) | 36 (25.7) | .998 ^a^ | 30 (18.0) | 23 (17.6) | 8 (19.0) | .833 ^a^ |
|  | DT physical symptom count (0-21), M (SD) | 10.3 (3.62) | 10.1 (3.77) | 10.9 (3.25) | **.044** ^c^ | 8.2 (3.78) | 8.1 (3.79) | 8.7 (3.77) | 0.331 ^c^ |

Abbreviations: SPC, specialist palliative care; O-SPC, outpatient specialist palliative care; I-SPC, inpatient specialist palliative care; DT, Distress Thermometer

^a^ Chi^2^-Test; ^b^ Fisher’s Exact Test; ^c^ T-Test (two-sided)

Significant group differences are marked in bold.
